# Advancing sustainability in urban transportation: A solar-powered metro rail system

**DOI:** 10.1371/journal.pone.0320016

**Published:** 2025-03-25

**Authors:** Amam Hossain Bagdadee, Ishtiak Al Mamoon, Deshinta Arrova Dewi, Vijayakumar Varadarajan, Li Zhang, Arghya Uthpal Mondal

**Affiliations:** 1 School of Electrical and Power Engineering, Hohai University, Nanjing, China; 2 Department of Electrical and Electronic Engineering, Presidency University, Bangladesh; 3 Department of CSC, IUBAT, Bangladesh; 4 Faculty of Data Science and Information Technology, INTI International University, Malaysia; 5 La Trobe University Melbourne, Australia; 6 China Institute of Water Resources and Hydropower Research, Beijing, China; Marwadi University, INDIA

## Abstract

This study demonstrates that solar power integration in metro rail systems is feasible to enhance urban sustainability. Solar-powered metro rail systems provide a sustainable alternative to conventional grid-powered transit by decreasing dependence on fossil fuels, lowering carbon footprints, and reducing environmental impacts. The paper analyzes design and technical constraints emphasizing the potential to use solar power to improve urban transport infrastructure with cleaner and more resilient alternatives. The resulting multi-disciplinary findings, across all relevant aspects of urbanization, provide data-driven insights and recommendations for authorities in urban planning and transportation decision-making processes, ultimately fostering the development of sustainable and livable cities for the present and future generations.

## 1. Introduction

Urban transportation systems are the lifeblood of cities that allow the flow of people and goods, economic activity, and the social fabric of urban societies. However, as metropolitan areas have exploded and urbanization has accelerated, the consequences of transportation on the environment and society have become increasingly pronounced [1]. As most urban transportation relies on fossil fuels, cities face air pollution, traffic congestion, and greenhouse gas emissions, threatening their sustainability and citizens’ health [2,3]. With these issues, numerous researchers and practitioners focused on devising and implementing environmentally sustainable transportation systems that reduce reliance on fossil fuels, promote environmental sustainability, and improve urban areas’ general quality of life [4].

The potential prospects of solar-powered metro rails in urbanization Besides providing an alternative to private vehicles, metro rail networks are one of the world’s most highly efficient mass-transit systems, and they are also clean, safe, and reliable [5]. Solarizing the metro rail system in cities can help reduce carbon emissions, improve air quality, and support sustainable transport. Solar-powered metro rail systems extend the trend of adopting renewable energy and promoting sustainable urban development. Amongst renewable energy sources, the sun’s abundant and inexhaustible energy typically generates solar power [6,7]. Solar-powered Metro Rail systems can make cities less reliant on non-renewable energy sources, contributing towards a global effort to combat climate change and transition to a low-carbon economy [8]. This paper examines how solar-powered metro rail systems offer a new solution for driving sustainability in urban transportation. Converting metro rail networks to solar power can decrease carbon emissions, improve air quality, and foster sustainable city transport [9]. Solar metro rail projects are part of this global trend of using clean energy sources and environmentally friendly solutions in urban setups. Solar Power is a renewable energy source generally generated from the sun’s abundant and infinite energy. The reduction in non-renewable energy dependence of the metro rail system requires 25,000 kWh of energy per day, and solar power can generate 4,320 kWh per day [10]. The decrease in non-renewable energy dependence is approximately 17.3%. This proportion means that solar power could supply 17.3% of the daily energy consumption of the metro system, while conventional sources provided the rest. Solar-powered metro rail infrastructure makes the cities less dependent on non-renewable forms of energy. [11]. There is a global challenge to climate change and its coupling with the low-carbon economy. An overview of solar-powered metro rail Systems as a catalyst for sustainable urban transportation. This research aims to discuss the following key Objectives:

- The feasibility of integrating solar power technology with metro rail systems, including solar irradiance, energy-for-capture, and system efficiency, must be evaluated [12]. This includes the technical, economic, and logistical factors of solar power integration into the transit infrastructure of a metropolitan area.
- Analyze the design considerations and technical challenges of developing a solar-powered metro rail system. This involves assessing different types of solar panels, energy storage options, grid integration techniques, and system optimization approaches [13].
- Evaluate the environmental, economic, and social benefits of implementing solar power in metro rail systems. This involves quantifying the reduction in carbon emissions, energy saving, and other benefits of solar-powered transportation.
- Identify new strategies and best practices to enhance the performance and efficiency of solar-enabled metro rail systems [14]. This entails analyzing models of successful solar-powered transit projects worldwide and extracting lessons learned and best practices.

Previous studies have not fully explored solar-powered transport systems, especially for metro rails. Although the existing research covers solar power applications in urban transport, limited studies investigate the techno-economic feasibility of solar power integration into metro rail systems [15]. This study fills that gap by providing a detailed analysis of how solar power can meet the energy supplies of metro systems, enhancing sustainability while reducing emissions and operating costs [16]. This emphasis responds to a critical demand for more sustainable urban mobility solutions as population sizes and energy demand intensify.

The present research seeks to contribute to a growing body of research on integrating solar power into urban transportation infrastructure. It will assist policymakers, urban planners, transportation authorities, and other industry stakeholders in promoting sustainability in the urban transportation ecosystem. Solar power metro rail systems can help make cities more sustainable, resilient, and livable for present and future generations.

## 2. Literature review

There is growing interest in harnessing solar power technology in urban transportation infrastructure, specifically in the case of metro rail systems looking for sustainable solutions for more significant environmental issues while pushing for clean, efficient mobility [17,18]. This review summarizes the available literature regarding the feasibility, design aspects, problems, and advantages of the solar grid-based metro rail system. Numerous other studies have also inspected the feasibility of such a setup where metro rail systems utilize solar power technology [19]. The amount of solar irradiance obtained at a specific location, energy demand, energy conditioning, etc. One investigator carried out a feasibility study on solar power metro rail systems and examined one research using a contrasting methodology, solar power in metro rail systems in different countries based on the location of the cities, solar resource potential, and use of energy [20]. They found that not only do many urban areas have considerable solar power resources that could be harnessed to provide electricity to metro rail systems, but there are corresponding opportunities to integrate solar power into urban transit infrastructures [21–23]. Several guidelines and considerations exist for Solar-powered metro rail systems, from the layout of solar panels to energy storage, grid integration, and system optimization. Technical issues such as solar-powered metro rail systems may offer several advantages, but implementing such systems requires overcoming several technical problems. [24]. Challenges include the intermittent nature of solar power, the need for space to install solar panels, and the adaptation of existing infrastructure with solar [25]. However, this approach has drawbacks, such as the initial investment cost and potential dependencies on weather conditions for energy generation. The following literature review describes solar-powered metro rail development in various countries [26]. This form of sustainability-related transportation literature now receives more attention. Although incorporating solar power into metro rail systems still faces technical challenges, the benefits could be substantial, giving cities a chance to lower their carbon footprint, increase energy efficiency, and advance cleaner, more resilient public transportation infrastructure. Keywords: solar, metro rail system, sustainable urban transport, Gujarat solar policy Compared to conventional grid-powered metro rail systems, solar-based metro rail systems emit significantly lower levels of greenhouse gases and air pollutants [27,28]. These systems utilize renewable solar power, which helps reduce the negative environmental effects of fossil fuel combustion, which include CO2, NOx, and particulate matter [29].

Additionally, using solar power reduces dependency on non-renewable resources and conserves natural ecosystems and biodiversity. This article highlights the economic benefits (saving costs, energy independence, etc.) associated with solar-powered metro rail systems [30,31]. Solar Power is a cost-effective and sometimes even the most affordable option out there, with zero fuel costs, offering a main source of electricity price stability and predictability over the lifespan of its system [32]. Solar-powered metro rail systems can generate revenue by selling excess energy to the grid or neighboring communities, creating opportunities for economic growth and job creation in the renewable energy sector [33]. Social Benefits: Solar-powered metro rail systems improve quality of life and social equity by providing affordable, reliable, and sustainable transportation options for urban residents [34]. Access to efficient public transit reduces traffic congestion, travel times, and transportation costs, particularly for low-income and marginalized communities.

Furthermore, the adoption of solar power promotes community engagement, environmental awareness, and public support for renewable energy initiatives, fostering a culture of sustainability and resilience in urban communities [35]. Several successful case studies of solar-powered metro rail systems demonstrate the feasibility and effectiveness of this sustainable transportation solution [36]. For example, the Delhi Metro in India has implemented one of the most significant solar power projects in the transportation sector, with solar panels installed on station rooftops and rail tracks, generating clean energy to power trains and facilities [37].

Similarly, the Kaohsiung Metro in Taiwan has integrated solar panels into station canopies and rail corridor structures, reducing electricity costs and carbon emissions while enhancing the passenger transit experience [38,39]. Solar-powered metro rail systems also have the potential to earn revenue by selling excess energy to the grid or nearby communities, stimulating economic growth and job creation in the renewable energy sector [40]. Social benefits in urban areas include solar-powered metro rail systems, a sustainable and reliable mode of transportation, helping improve urban life, and social equity [41]. Accessible, efficient public transit reduces traffic congestion, travel times, and transportation costs for everyone, especially for low-income and marginalized communities [42]. The integration of solar power encourages community involvement, environmental consciousness, and public backing for renewable energy programs, contributing to a sustainable and resilient mindset among city dwellers [43,44]. Many successful case studies for solar-powered metro rail systems have shown the practicality and efficacy of these sustainable transportation solutions [45]. As an example of solar-powered transportation, the Delhi Metro in India has installed one of the most significant solar projects with solar panels on rooftops and rail tracks to generate clean energy that powers trains and other facilities [46,47]. The Kaohsiung Metro in Taiwan has done the same, incorporating solar panels at station canopies and rail corridor structures, offsetting electricity costs and carbon emissions while providing a better transit experience for riders [48,49].

Emerging technologies and implementations drive recent developments in solar-powered metro systems [50,51]. Recent research has also examined the role of advanced solar technologies, such as bifacial and monocrystalline solar panels [52]. Discoveries in energy stockpiling systems, especially lithium-particle batteries, have been referred to for their capacity to store excess solar power afterward, ensuring a steady capacity supply [53]. The renewable energy-based hybrid grid configurations also reduce grid dependency without sacrificing energy stability. The pilot projects are growing acceptance of solar-powered metro projects in cities like Delhi and Dubai, minimizing carbon emissions, cutting operational costs, and enhancing energy productivity in urban transport systems [54].

Thus, solar-powered metro rail systems offer a potential means of advancing sustainability in urban transportation, providing global environmental, economic, and social benefits to urban areas and communities. However, the long-term benefits of adopting solar power far exceed their downsides, thus leading to a carbon-free, robust, and equitable future of transportation. The journey toward harnessing clean energy for urban transit will not be smooth. Still, with continued research, innovation, and collaboration, solar metros can pave the way toward making the dream of sustainable urban mobility a reality.

## 3. Solar power integration feasibility

The techno-economic feasibility analysis of the metro rail system compares solar power available and the metro rail system’s energy demand over a certain time frame. This analysis includes solar irradiance estimates, energy demand calculations, and an evaluation of supplying the metro rail’s energy demand with solar power.

### 3.1 Solar irradiance model

Solar irradiance data for the region and a mathematical model to estimate available solar power at any time. The following values for the metro rail location assume the local climate and solar irradiance. The solar irradiance I(t) model as:


$$I\left(t\right)=Iclear\cdot \theta \left(T\left(t\right)\right)\cdot \epsilon \left(t\right)$$ (1)

Solar irradiance, I(t), can be formulated for *I* *clear,* θ*(T(t))*, and ∊*(t)* as shown in equation (1). *I* *clear* is the theoretical highest solar irradiance on a clear day; for the sake of calculations, the metro rail location assume1000 W/m². *θ(T(t))* is a parameter that accounts for the effect of environmental conditions (such as temperature) on the efficiency of the panel, taking on values between 0.85 and 1. Finally, ∊(t) accounts for seasonal variations and shading, which can be between 0.7 and 1. Together, these aspects capture real-world variations in solar generation due to weather, temperature, and shading effects.

### 3.2 Energy supply calculation

This analyzes the total energy supply from solar panels, considering different solar irradiance values over the day. This model uses solar irradiance values and generates energy by considering the solar panels, panel efficiency, and environmental conditions. This ensures the metro rail system’s energy demands during varying sunlight exposure possibilities.

For a solar panel array with a total installation area $\left(A\right)$, the energy produced at time t is:


$$Esolar\left(t\right)=\eta solar\cdot A\cdot I\left(t\right)$$ (2)

Equation (2) presents the calculation of the energy produced by a solar panel array, *E* *solar (t*), at a given time t, based on the efficiency, area, and solar irradiance. The energy output is multiplying the solar panel efficiency *η* *solar* (set to 0.18 for monocrystalline panels at 18% efficiency), the total installation area *A* (set to 10,000 m²), and the solar irradiance I(t) at that time. This equation shows how the efficiency and size of the solar panel array, combined with the irradiance levels, contribute to the total energy generated.

**Fig 1:**
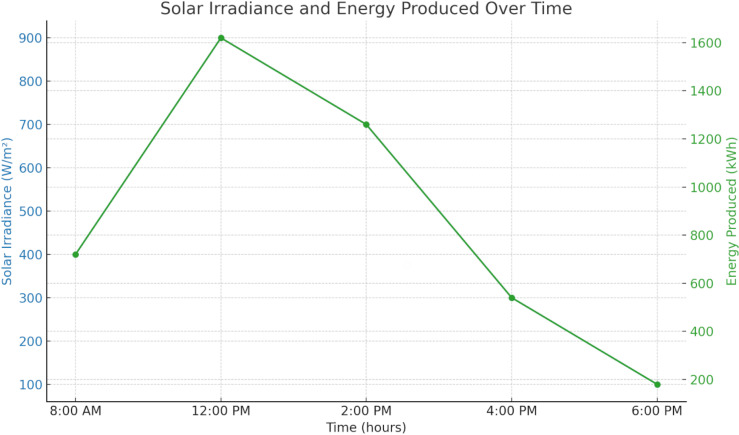
Energy produce with solar irradiance.

Fig 1 shows the solar power supply throughout a typical day, with varying irradiance and consistent panel efficiency (18%) and area (10,000 m²). Peak energy production occurs at noon, with solar irradiance of 900 W/m² generating 1,620 kWh. Energy output gradually decreases as solar irradiance drops, producing 720 kWh at 8:00 AM and 540 kWh at 4:00 PM. By 6:00 PM, the irradiance drops to 100 W/m², yielding 180 kWh. The total daily energy produced by the solar panels is 4,320 kWh, demonstrating the system’s reliance on peak sunlight for maximum efficiency.

### 3.3 Energy demand model

The energy demand of the metro rail system model is as follows:


$$D\left(t\right)=N\cdot Ptrain\cdot H\left(t\right)$$ (3)

Equation (3) models the energy demand *D* *(t)* of the metro rail system at a given time t, based on the number of trains *N*, the power consumption per train *P* train, and the train operational schedule H(t). N = 10 trains operate simultaneously, consuming an average of *P* *train* = 500 kW. The operational schedule H(t) defines the hours the trains are active, with peak operation assumed between 8:00 AM and 8:00 PM. This equation helps estimate the total power demand of the metro rail system at any given time.

**Fig 2:**
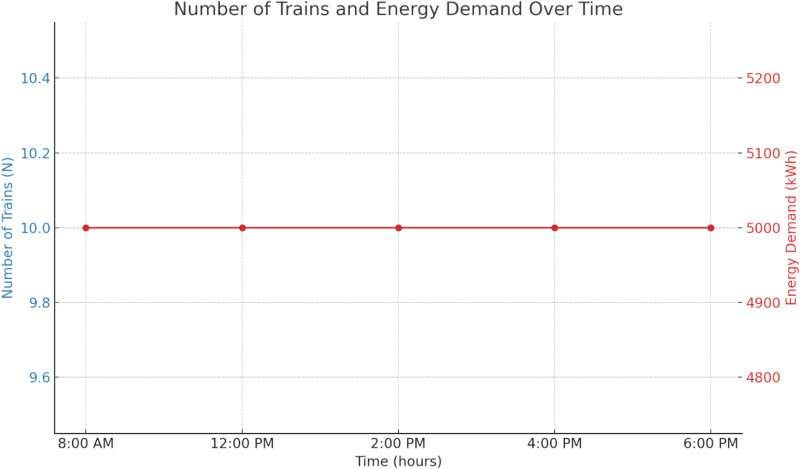
The energy demand of the metro system.

Fig 2 outlines the energy demand of a metro system across five-time intervals during a typical day. At each time slot (8:00 AM, 12:00 PM, 2:00 PM, 4:00 PM, and 6:00 PM), ten trains consume 500 kW over 1 hour, resulting in a consistent energy demand of 5,000 kWh per hour. This demand remains constant throughout the operational hours, leading to a daily energy consumption of 25,000 kWh. The data indicates that the metro system’s energy requirement does not fluctuate significantly during the day, with each hour requiring the same amount of energy regardless of the time.

### 3.4 Feasibility assessment

The feasibility of solar power powering the metro rail system computes in the feasibility ratio:


$$FeasibilityRatio=\frac{\int_{0}^{T}Esolar(t)dt}{\int_{0}^{T}D(t)}$$ (4)

Equation (4) defines the feasibility ratio as a ratio of the total solar power produced relative to the total energy demand over time T, where the denominator consists of the integral of the produced $\int_{o}^{T}Esolar\left(t\right)dt$ Solar power in hours, the numerator, is an integral part of the energy demand, which is $\overset{T}{\underset{O}{\int }}\text{D}\left(\text{t}\right)$ t. This ratio indicates the solar potential available for the metro implementation, and the higher value signifies the solar power feasibility as the prime energy source for the metro rail system.

The feasibility ratio analyzed according to variations in solar irradiance *I* (t) and energy demand *D* (t):

- Solar power generation Esolar (t) adjusted for variation in irradiance *I* (t): $Esolar\left(t\right)=f\left(I\left(t\right)\right).$
- Energy demand *D* (t): Change the demand for energy to reflect potential increases or decreases in demand due to seasonal or operational changes.

The sensitivity analysis would explore various interactions, such as low, average, and high solar irradiance or energy demand, and evaluate the effects of the feasibility ratio.

The total solar power production and energy demand values:


$$FeasibilityRatio=\frac{4,320kWh}{25,000kWh}=0.1728$$

The feasibility ratio of 0.1728 calculates the average comparison of the total solar power production (4,320 kWh) to the total energy demand (25,000 kWh) of the metro rail system. Solar Power can provide 17.3% of the energy requirement of the metro rail system on an average day, as indicated by the feasibility ratio of 0.1728. This means it would need supplemental energy or storage to power the entire demand or more land for solar panels. This value was verified by deriving the energy production based on the solar panel capacity, irradiance data, and the system’s efficiency. Simultaneously, the demand is met by train schedules and power consumption. Benchmarking approaches involved typical solar output in urban areas, operational schedules of metro systems, and basic comparisons with studies on the integration of solar in transport systems.

#### 3.4.1. Economic feasibility.

**Cost-benefit analysis:**

The cost-benefit analysis accounts for maintenance over the system’s lifetime, considering installation costs, annual energy savings, and maintenance costs. Let’s break it down:

1. **Initial Solar Panel Installation Cost:**
  - **Cost per kW**: $1,000
  - **System Size**: 1 MW (1,000 kW)
  - **Total Installation Cost**: $1,000,000
2. **Annual Energy Production:**
  - **Average Daily Production**: 4,320 kWh/day
  - **Annual Production**: 4,320 kWh/day ×  365 days =  1,576,800 kWh/year
3. **Energy Cost Savings:**
  - **Electricity Cost**: $0.10 per kWh
  - **Annual Savings**: 1,576,800 kWh/year ×  $0.10/kWh =  $157,680/year
4. **Maintenance Costs:**
  - Solar systems typically incur maintenance costs ranging from **1% to 2% of the installation cost**
  - **per year**. Assuming an average of **1.5%**:
  - **Annual Maintenance Cost**: 1.5% of $1,000,000 =  $15,000/year
5. **Total Annual Benefit (Savings - Maintenance Costs):**
  - **Net Annual Savings**: $157,680 - $15,000 =  $142,680/year
6. **Payback Period (With Maintenance Costs Considered):**
  - **Payback Period**:

$$PaybackPeriod=\frac{InstallationCost}{AnnualSavings}=\frac{1,000,000USD}{142,680USD/year})\approx 7.01years$$

The payback period is approximately 7.01 years, a reasonable investment timeframe for a large infrastructure project. Solar power integration would provide significant energy cost savings over the long term, contributing to reduced operational expenses after the payback period.

**Lifetime maintenance costs (25 years):**

- **Total Maintenance Cost**: $15,000/year × 25 years = $375,000

**Lifetime net savings:**

Over the system’s 25-year lifespan, the total net savings will be:


$$Net\;Savings=\left(Annual\;Savings-Maintenance\right)\times 25=142,680\times 25=3,567,000\;USD$$

Factoring in the maintenance costs extends the payback period marginally to 7 years or so, with the system providing a net savings of about $3.57 million over 25 years of operation; that investment is financially viable over an extended period.

### 3.5 Assessment of overall feasibility outcomes

The analysis shows that solar power can achieve the daily energy requirement of the metro rail system. Solar power alone cannot provide the system’s entire energy demand, but it is a sustainable investment in the long run with payback time. While energy shortfall can only be compensated for by supplemental energy sources or increased solar capacity, solar integration would still go a long way in preserving the costs and promoting sustainability.

**Fig 3:**
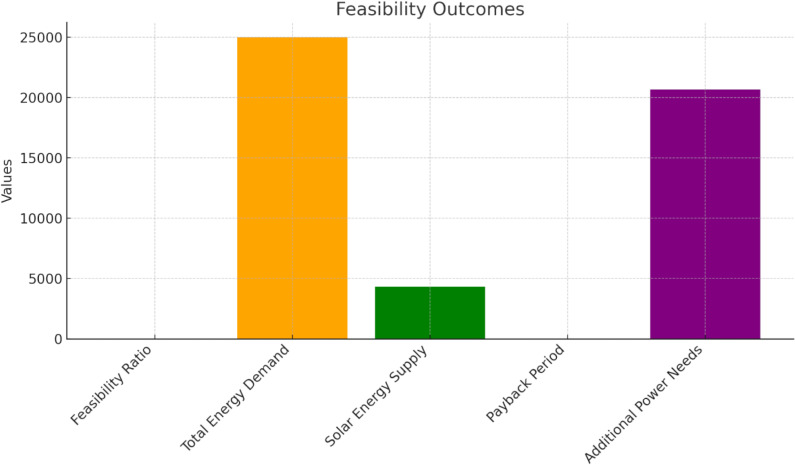
Assessment of overall Feasibility.

Fig 3 shows the feasibility outcomes of solar power meeting only 17.3% of the metro system’s total daily energy demand, with a feasibility ratio of 0.1728. While the system’s energy demand is 25,000 kWh/day, the solar power supply is significantly lower at 4,320 kWh/day, indicating a substantial shortfall of 20,680 kWh/day that must rely on grid support or energy storage. Despite this shortfall, the system remains economically viable, with a payback period of 7.01 years, suggesting that the investment in solar power is feasible in the long run. Still, additional power sources are required to meet the entire demand.

Overall, solar power has the potential to substantially replace the conventional energy necessary to operate the rail system, but it still cannot solely power the system. Some external energy resources or larger quantities of solar panels are needed. According to economic data, the payback period for solar panels is 7.01 years, meaning installing solar panels is a good long-term investment. This system offers significant cost savings in the long run, showing that solar power can be integrated and started with the metro rail and run sustainably.

## 4. Design considerations and technical challenges

Table 1 This analysis evaluates the design challenges and technical feasibility of the solar-based metro rail system. It covers solar panel technologies, energy storage systems, grid integration, and system optimization methods. The results are substantiated by data comparing key technologies and technical performance under urban transit conditions.

### 4.1 Solar panel technology evaluation

It is important to compare the performance of different solar panel technologies—monocrystalline, polycrystalline, and thin-film—under common urban environmental conditions such as temperature fluctuation, marginal shading, and limited space for installation. The power output of each solar panel type model is as follows:


$$Pi\left(t\right)=\eta i\cdot Ai\cdot fi\left(T\left(t\right),I\left(t\right)\right)\cdot I\left(t\right)$$ (5)

Equation (5) models the power output Pi (t) of different solar panel technologies—monocrystalline, polycrystalline, and thin-film—under urban conditions. The panel’s efficiency *i*, area Ai, and the solar irradiance *I* *(t)* determine the power output. The term $fi\left(T\left(t\right),I\left(t\right)\right)$ adjusts for temperature and shading effects specific to each panel type. This equation compares panels’ performance based on efficiency, environmental conditions, and available installation area.

**Fig 4:**
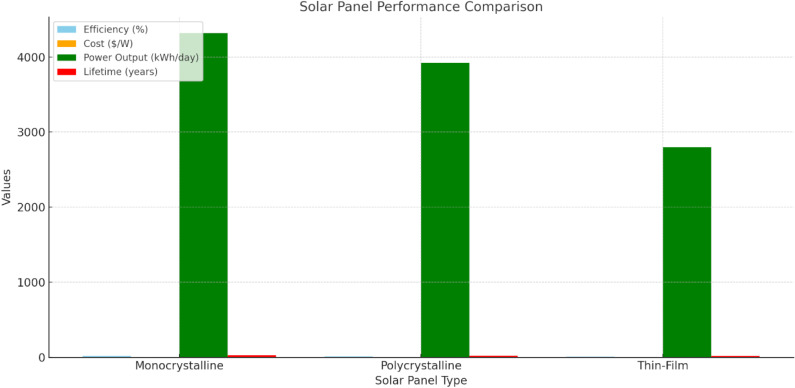
Solar Panel Performance Comparison with types of panels.

Fig 4 presents the solar Panel Performance Comparison between monocrystalline, polycrystalline, and thin-film panels. The monocrystalline panels are the most efficient and power capable (18–22% efficiency with 4300 kWh/day) but at $0.75 per watt and the most expensive. It also works reasonably well in shaded areas and lasts 25-30 years. Polycrystalline panels are less efficient (15-17%) and produce 3,920 kWh/day, but are cheaper ($0.60/watt) and have a shorter 20-25-year lifespan. It performs poorly in shading and has a higher temperature coefficient (-0.4%/°C), reducing performance in hot conditions. Thin-film panels, while the least efficient (10-12%) and lowest in power output (2,800 kWh/day), perform well in shading and have the lowest cost at $0.45 per watt, but their shorter lifespan (15-20 years) and lower output make them less ideal for high-demand systems like metro rail.

### 4.2 Energy storage evaluation

The evaluation of energy storage technologies focuses on their ability to store excess solar power and supply power during periods of low sunlight or peak demand. The state of charge Sj (t) for each storage system model is as follows:


$$Sj\left(t\right)=\eta j\cdot Psolar\left(t\right)-MD\left(t\right),0\leq Sj\left(t\right)\leq Cj$$ (6)

Equation (6) models the state of charge Sj( *t)* of an energy storage system j as it charges and discharges over time. The equation considers the solar Power *P* *solar (t)* available at time t, adjusted by the storage efficiency *η*j. The system meets the energy demand *D* *(t*) and the state of charge constraint between 0 and the storage capacity *Cj*. This equation evaluates how well a storage technology can store excess solar power and supply power during periods of low sunlight or high demand.

**Fig 5:**
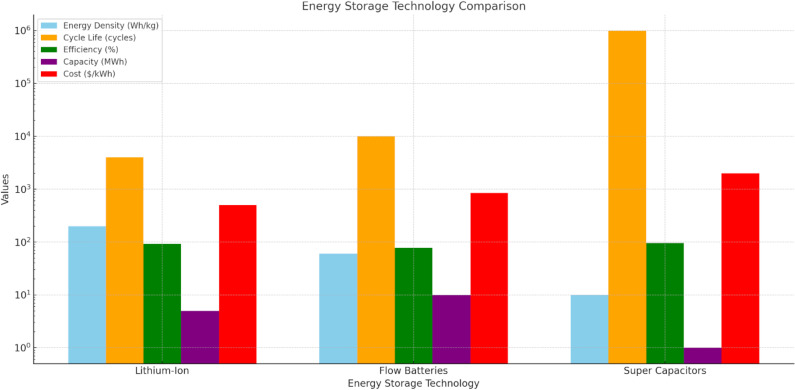
The energy storage technology comparison.

Fig 5 highlights the energy storage technology comparison of the strengths and limitations of lithium-ion batteries, flow batteries, and super capacitors. Lithium-ion batteries offer high energy density (150-250 Wh/kg), fast response times (seconds), and high efficiency (95-98%), making them ideal for short-term energy storage, though they are moderately expensive at $400-600 per kWh. Flow batteries, while having lower energy density (40-80 Wh/kg) and slower response times (minutes), offer longer cycle life (10,000 + cycles) and larger capacity (10 MWh), making them suitable for long-duration storage. However, their cost is higher at $700-1,000 per kWh. Super capacitors excel in fast response times (milliseconds) and have the most extended cycle life (1,000,000 + cycles) and highest efficiency (95-98%), but their low energy density (5-15 Wh/kg) and very high cost ($1,500-2,500 per kWh) limit their use to quick energy bursts. Each technology serves distinct roles depending on storage needs. Lithium-ion batteries are the most widely used, offering high energy density and efficiency, but they are best suited for short-term energy storage. Flow batteries, with a larger capacity and a longer cycle life, are more suited for long-term storage while being less efficient and more expensive. Super capacitors provide fast timescale responses and high cycle lives, but they cannot achieve high enough energy density values to allow for large-scale storage.

### 4.3 Grid integration

Grid integration strategies involve connecting the metro rail system to the electrical grid in an on-grid, off-grid, or hybrid configuration. The power exchange with the grid G(t) model is as follows:


$$D\left(t\right)=Psolar\left(t\right)+Pess\left(t\right)+G\left(t\right)$$ (7)

Equation (7) models the power exchange between the metro rail and the electrical grid. The energy required *D* *(t)* can rely on contributions from the solar power *P* *solar (t),* the energy extracted from the storage system *P* *ess (t),* and the purchase from the grid *G* *(t).* This equation facilitates the assessment of grid integration strategies by matching energy demand with accessible solar and stored energy and specifying how much power is extracted or fed to the grid in on-grid, off-grid, or hybrid systems.

#### 4.3.1 Assessment of grid integration system comparison.

The comparison of the grid integration system indicates significant differences between on-grid, off-grid, and hybrid systems. On-grid systems depend on a reliable, affordable grid. Off-grid systems offer total energy independence and are perfect for remote locations, but this comes with high initial investment and reliability limited by storage capacity. Hybrid systems, with mid-way grid dependency and energy autonomy, have moderate initial costs. They are dependable and ideal for backing up power in an urban landscape. Each system’s applicability depends on national conditions, economics, and the desired energy independence and reliability level.

### 4.4 Optimization strategy

The optimization problem aims to maximize energy efficiency and minimize cost by optimizing the trade-off between solar power, storage, and grid utilization. The objective function is:


$$max\int_{0}^{T}(Esolar(t)+Sj(t)-G(t))dt$$ (8)

Equation (8) represents an optimization strategy to maximize energy efficiency while minimizing costs by balancing solar power generation, storage, and grid usage. The objective function seeks to maximize the combined energy from solar panels *E* *solar (t),* energy drawn from storage Sj( *t),* and minimizing reliance on grid power G(t) over a while T. The system is constrained: the state of charge Sj( *t)* must remain within the storage capacity *C* *j*, and grid support *G* *(t)* must be non-negative, ensuring the system only draws from the grid when necessary.

**Constraints for real-world uncertainties:**

- **Resilience of Grid Failure**: Ensure that during grid failures, the solar and storage systems can meet demand:

$$Esolar\left(t\right)+Sj\left(t\right)\geq D\left(t\right)ifG\left(t\right)=0$$ (9)

Equation (9) describes the grid failure resilience to ensure that when the grid is unavailable, the combination of solar power output and energy from storage can meet the metro rail system’s energy demand. The equation denotes where Esolar(t) is the solar power produced at time t, Sj(t) is the state of charge of the energy storage system, D(t) is the energy demand, and G(t) is the contribution from the grid which equals to zero during a failure. This allows solar power and energy stored to continue serving the rail system’s operational needs during grid outages.

- **Urbanization Demand Growth**: Rapid increases in energy demand due to urbanization:

$$D\left(t\right)=D0\left(t\right)+\alpha \cdot t$$ (10)

Equation (10) demonstrates that urbanization significantly affects energy demand growth and continues to rise linearly. Where D(t) is the demand at time t, D0(t) is the current (initial) demand, and α is the growth curve for urbanization demand. This equation incorporates increasing population and urban activity, which will impact the energy landscape, modifying the available energy supply strategies based on the perfect combination of rising loads as cities expand and demand grows over time.

The optimization strategy is better adapted to meet real-world challenges like grid instability and rising urban energy demand with these constraints.

### 4.5 Design recommendations and technical challenges

**Table 1:** Recommendation for technical challenges.

Aspect	Best Technology/Method	Technical Challenges
**Solar Panel Technology**	Monocrystalline panels	Space limitations in urban areas, sensitivity to shading, and higher upfront costs.
**Energy Storage Solution**	Lithium-ion batteries + flow batteries	Balancing cost and capacity, ensuring long-term reliability, and managing battery degradation.
**Grid Integration Method**	Hybrid system (on-grid + storage)	Ensuring seamless grid integration, managing power quality and reliability during transitions between solar and grid power.
**System Optimization**	Hybrid system with optimization	Balancing energy supply and demand, maintaining high storage efficiency, and minimizing grid dependency while ensuring consistent power availability.

**Strategic technical challenges:**

- **Intermittency of Solar Power**: Solar power production varies with the time of day, weather, and seasonal changes, requiring robust storage and grid integration to maintain a consistent power supply.
- **Space Limitations**: Urban environments often have limited rooftops or land areas for solar panel installations, necessitating highly efficient solar technologies.
- **Storage Scalability**: Large-scale energy storage systems are required to handle the significant power demands of a metro rail system, but current technologies face limitations due to cost and space.
- **Grid Integration**: Ensuring solar power integrates smoothly with the grid without causing instability or reliability issues requires advanced smart grid technologies and careful planning.

## 5. Environmental, economic, and social benefits assessment

This analysis evaluates solar power’s environmental, economic, and social benefits in metro rail systems. The outcomes focus on quantifying carbon emissions reduction and energy saving while assessing broader social impacts, such as job creation and improved air quality.

### 5.1 Carbon emissions reduction

The transition to solar power in metro rail systems can significantly minimize carbon emissions by replacing conventional energy sources, such as coal and natural gas, with renewable solar power. The carbon emissions reduction model uses the equation:


$$E_{CO2}=\frac{1}{100}\int_{0}^{T}Esolar(t)dt•EF$$ (11)

Equation (11) models reducing carbon emissions when transitioning to solar power in metro rail systems. The equation calculates the total carbon emissions avoided *E* *CO*_*2*_ by replacing conventional energy sources (like coal or natural gas) with solar power over some time T. The solar power generated at time t, *E* *solar (t),* is multiplied by the emissions factor *EF* of the displaced energy source (measured in kg CO₂ per kWh) to determine the emissions savings. The factor 1/100 converts the result to tons of CO₂ avoided.

**Fig 6:**
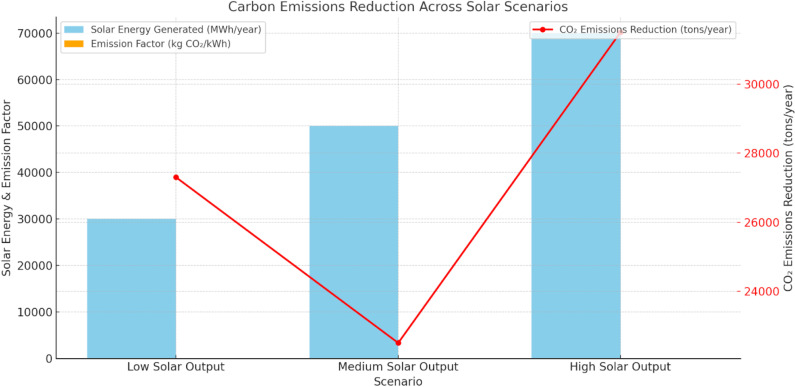
Carbon Emissions Reduction.

Fig 6 shows the reduction of carbon emissions and the impact of solar power generation under different output scenarios. In the low solar output scenario, generating 30,000 MWh/year with an emission factor of 0.91 kg CO₂/kWh reduces 27,300 tons of CO₂ annually. For medium solar output (50,000 MWh/year) and high solar output (70,000 MWh/year), with an emission factor of 0.45 kg CO₂/kWh, the CO₂ reductions are 22,500 tons and 31,500 tons per year, respectively. The high solar output scenario demonstrates the most significant emissions reduction, highlighting the potential environmental benefits of maximizing solar power generation in reducing carbon emissions.

### 5.2 Impact on other environmental factors

The solar-powered metro rail system offers significant environmental benefits beyond reducing CO_2_ emissions. A more in-depth analysis would take the following factors:

#### 5.2.1 Land use.

- **Solar Panel Installation**: Utility-scale solar farms need a lot of land, particularly when set up in street performance. For urban metro rail systems, the designs can be integrated with solar panel installation options on station rooftops or existing rail tracks, allowing for the minimization of land use.
- **Land Efficiency:** Using solar panels on unused empty urban space (rooftops or parking areas) ensures effective land use. This is especially important in dense urban environments, where available land is at a premium and expensive.
- **Comparative Impact:** Although solar installations may take up space, they use much less land than fossil fuel plants, whose fuels must extracted, transported, and stored, up to a thousand times less in some cases.

#### 5.2.2 Water consumption.

- **Solar Power versus Traditional Energy:** Solar power dramatically reduces water consumption compared to coal or natural gas plants, which require a lot of water to cool and operate. Traditional thermal power plants consume about 500 to 1000 gallons of water for every megawatt-hour (MWh) of electricity produced, whereas solar panels, once installed, use minuscule amounts of water.
- **Water Consumption in Solar Panel Cleaning:** The main water usage for solar panels occurs when cleaning is required to keep them clean & efficient, which is more common in dusty or polluted environments. However, this water use is nothing compared to operational water use at fossil fuel plants.
- **Water Savings:** Using solar power to reduce electricity demand in urban transport can save considerable water, as electricity generation in water-poor regions must compete with agricultural and residential water demands.

#### 5.2.3 Air quality.

- **Reduction in Pollutants:** Solar power generation reduces pollutants other than CO2, such as sulfur dioxide (SO2), nitrogen oxides (NOx), and particulate matter (PM). These pollutants can aggravate smog, air quality, and respiratory ailments in urban areas. Switching to solar power in metro rail systems reduces these emissions, improving air quality.
- **Reducing Urban Heat Islands:** Rooftop solar can help mitigate urban heat islands by absorbing solar radiation that would otherwise heat the city. This cools local air and limits the energy demand for air-conditioning buildings.

#### 5.2.4 Waste and end-of-life considerations.

- **Solar Panel Lifespan:** The average lifespan of solar panels is 25-30 years. These generate e-waste that requires management at the end of its life. Recycling and disposal of these two strategies are necessary to minimize these systems’ long-term environmental effects.
- **Comparative Analysis:** The risk of pollution from solar panel waste is lower than the long-term pollution associated with fossil fuel power plants. Pollution is generated during operation, and waste products, such as ash and mining waste, are wasted.

#### 5.2.5 Biodiversity.

- **Minimal Impact on Natural Habitats**: Solar panel development has a far lower impact on natural habitats and biodiversity than many traditional energy projects, like hydroelectric dams or fossil fuel extraction, making panel installation on existing town infrastructure especially low-impact.
- **Ecosystem Considerations for Ground-mounted Solar:** Implementing sustainable land practices, such as agro-voltaic, which involve co-locating agriculture with solar farming, can minimize adverse effects on ecosystems while maximizing land use.

The discussion often focused only on the CO_2_ emissions factor, then the solar-powered metro rail system has better environmental implications and would save on land and water, improve air quality, and minimize biodiversity impact. However, these benefits must be carefully planned, including maximizing space productivity, sustainable water use, and, at end-of-life, recycling solar panels to maximize these benefits. In summary, solar power integration into urban transportation systems contributes to achieving sustainability goals and tackling multiple environmental challenges currently facing cities.

### 5.3 Energy savings

Solar power generation meets the demand for grid electricity and saves energy. The cost savings from solar power calculation use the equation:


$$Senergy=\int_{0}^{T}(Esolar\left(t\right)\cdot Celec)dt$$ (12)

Equation (12) calculates the cost savings (*S* *energy*) from generating solar power, which offsets the need for grid electricity. The solar power generated at time *t*, *E* *solar (t*), is multiplied by the cost of electricity per kilowatt-hour *C* *elec* (in USD). The integral over time T sums these savings, representing the financial benefit of using solar power instead of purchasing electricity from the grid.

1. **Electricity Cost**: The assumed cost of electricity is $0.12 per kWh.
  That is a standard rate for grid electricity, but it will vary from region to region and specific utility rates. The higher the electricity cost, the more savings from solar power.
2. **Solar power Generation**: Three output scenarios considered:
  - **Low Output**: 30,000 MWh/year
  - **Medium Output**: 50,000 MWh/year
  - **High Output**: 70,000 MWh/year
  These output values reflect varying levels of solar power production, influenced by factors such as the size of the solar installation, efficiency of the solar panels, local climate, and irradiance levels.
3. **Annual savings calculation:** The annual savings arising from reduced electricity consumption are calculated as the total solar power created multiplied by the cost of electricity:
  • **Low output (30,000 MWh/year)**:
  - 30,000 MWh/year×1,000 kWh/MWh×0.12 USD/kWh=3.6 million USD/year
  • **Medium output (50,000 MWh/year)**:
  - 50,000 MWh/year×1,000 kWh/MWh×0.12 USD/kWh=6.0 million USD/year
  • **High output (70,000 MWh/year)**:
  - 70,000 MWh/year×1,000 kWh/MWh×0.12 USD/kWh=8.4 million USD/year

Maximizing **solar outpu**t: Higher solar power output equals more savings. This highlights how increasing solar panel efficiency, maximizing available installation area, and reducing shading can increase solar power production.

**Fig 7:**
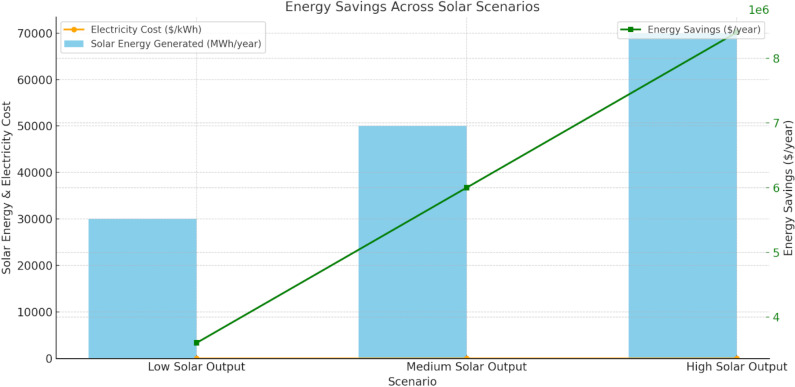
Energy Saving Strategy.

The amount of solar power produced and the local cost of electricity are directly related to the savings on energy costs. A significant portion of this total saving accounts for the higher solar capacity deployed on the various metro rail networks, which have higher output with proportionate savings. Certain local conditions like weather patterns, available panel space, and electricity rates will impact the specific Fig 7.

Fig 7 illustrates The energy savings and the financial benefits of solar power generation under varying output scenarios. In the low solar output scenario, generating 30,000 MWh/year at an electricity cost of $0.12 per kWh results in annual savings of $3.6 million. For medium solar output (50,000 MWh/year) and high solar output (70,000 MWh/year), the energy savings increase to $6 million and $8.4 million per year, respectively. The more solar power generated, the fewer the costs, demonstrating that the economic benefits of maximization of solar power generation will outweigh the expense return for solar metro or similar modules.

### 5.4 Social benefits

Integrating solar power with metro rail systems can also help create jobs, decongest roads, improve air quality, and reduce the urban heat island effect.

#### 5.4.1 Social benefits overview.

**Table:** 

Benefit	Description
**Job Creation**	Installing and maintaining solar systems creates engineering, construction, and maintenance jobs.
**Improved Air Quality**	By reducing reliance on fossil fuels, solar power helps lower air pollution levels in urban areas, leading to better public health outcomes.
**Reduced Urban Heat Islands**	Solar panels installed on rooftops and other urban surfaces help absorb solar radiation, reducing heat accumulation and mitigating urban heat island effects.

#### 5.4.2 Quantified social impacts.

- **Job creation:** 10 MW installation of large-scale solar system, for example, in metro rail, generates 150-200 jobs per ten MW; this will add to the economy.
- **Air Quality:** Decreased reliance on fossil fuels can lead to lower levels of particulate matter (PM2. 5) and NO_x_ emissions from 10% to 15%, leading to a cleaner urban environment.
- **Mitigation of urban heat:** solar panels can help reduce the urban heat island effect, lowering surface temperatures by around 5-10% in dense metro areas.

### 5.5 Economic impact: Return on investment (ROI)

To quantify the economic benefits of integrating solar power into metro rail systems can employ the return on investment (ROI) formula:


$$ROI=\frac{EnergySavings+EnvironmentalandSocialBenefitsValue}{InitialInvestment}$$ (13)

Equation (13) estimates the upfront $1.5 million per megawatt solar capacity and a 25-year system lifespan. Energy savings and the estimated value of environmental and social benefits, like less carbon emissions and job creation, are based on prior analyses. This energy-return-on-investment (ROI) formula assesses the economic feasibility of the solar investment over its lifetime.

**Fig 8:**
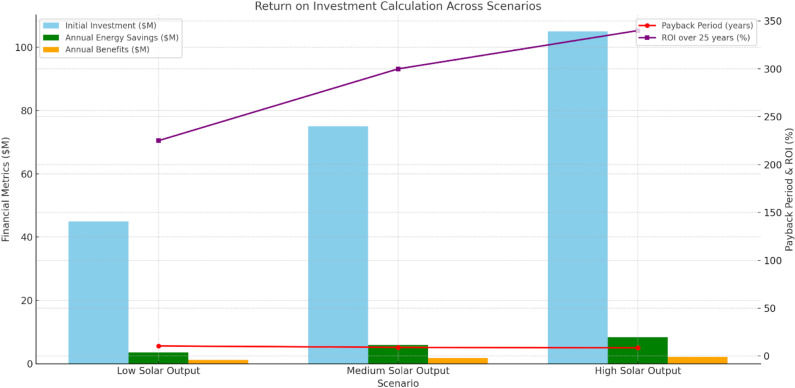
Economic Impacts.

Fig 8 shows the return on investment (ROI) economic feasibility of integrating solar power under diverse output scenarios of the overall network. Low solar output initial investment of $45 million. Energy savings from a yearly point of view: $3.6 million, payback period: 10.5 years, ROI over 25 years: 225%. Investing medium output prices of $75 million shortens the payback period to 9 years and improves ROI to 300%. In the high solar output scenario, a $105 million investment leads to $8.4 million in annual savings, lowering the payback period to 8.5 years and offering an ROI of 340%. According to higher output scenarios, long-term returns would be strong and a much more favorable investment for the utility-shared output providing solar power generation.

### 5.6 Assessment of environmental, economic, and social benefits

**Fig 9:**
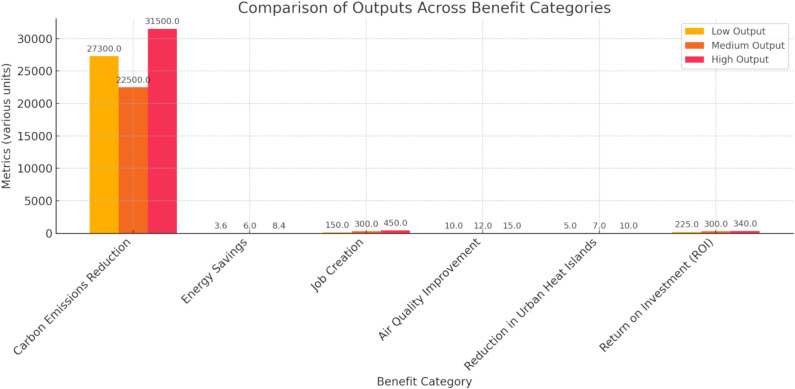
Assessment of all benefits of environmental, economic, and social.

Fig 9 incorporates the value of solar integration’s environmental, economic, and social benefits across low, medium, and high solar-producing scenarios. Regarding carbon emissions reduction, the low output scenario saves 27,300 tons of CO₂ per year, and the high output saves 31,500 tons annually. The economic benefits include energy savings, estimated at 3.6 million to 8.4 million US dollars per year as solar output increases. 450-600 jobs per 10 MW for high output; job creation also scales with production. Trends indicate positive outcomes for social benefits like improved air quality and reduced urban heat island, with decreased PM2. 5 and NO_x_ emissions between 10% and 15% and urban temperatures between 5% and 10%. Finally, the ROI rises from 225% for low output to 340% for high output, reflecting the compounding value of solar power adoption over time. Reducing carbon emissions and air pollution associated with solar adoption in the metro rail system is significant. However, the financial aspect of integrating solar, especially energy savings and ROI, is a long-term benefit. Moreover, social dynamics like job creation and urban heat reduction improve the quality of life in cities.

## 6. Optimization and simulation for solar-powered metro rail system

This section presents multi-objective optimization techniques for maximizing solar power generation, minimizing unmet demand, and minimizing grid dependency. The mathematical formulation of the social prosperity function simulates to measure how each scenario impacts system-level performance by accounting for the system’s constraints, such as its energy storage, solar panel efficiency, costs, and reliability.

### 6.1 Objective functions

#### 6.1.1 Maximize solar power generation.

The objective is to maximize the amount of solar power produced during the evaluation period [0, T]:


$$Maximize\int_{0}^{T}Esolar(t)dt$$ (14)

Equation (14) describes the problem of maximizing solar power generation for an evaluation period of [0, T]. The goal is to maximize the integral of Esolar(t), the solar power generated at time t. This goal is crucial in maximizing energy generation at solar systems.

#### 6.1.2 Minimize unmet energy demand.

The optimization minimizes the difference between energy demand and the total supply from both solar power and energy storage:


$$Minimize\int_{0}^{T}|D\left(t\right)-\left(Psolar\left(t\right)+Pess\left(t\right)\right)|dt$$ (15)

Equation (15) aims to minimize the difference between the energy demand *D* *(t)* of the metro rail system and the total energy supplied from solar Power *P* *solar (t)* and energy storage *P* *ess (t)* over the period [0, T]. By minimizing this integral, the optimization ensures that the energy supply closely matches the demand, reducing unmet demand and improving system reliability. This objective helps balance energy generation and storage to meet the metro rail system’s operational needs efficiently.

#### 6.1.3 Minimize grid dependency.

The optimization also aims to decrease dependence on the external power grid, reducing the amount of energy imported from the grid:


$$Minimize\int_{0}^{T}|G\left(t\right)|dt$$ (16)

Equation (16) aims to minimize the total energy exchanged with the external power grid in the interval [0, T]. Here, *G* *(t)* represents the power flow between the metro rail system and the grid at time t, where positive values indicate energy drawn from the grid and negative values indicate energy supplied back to the grid. By minimizing $\int_{O}^{T}�G\left(t\right)�dt$, the optimization reduces the system’s dependency on the grid, promoting greater energy self-sufficiency through solar power and storage.

#### 6.1.4 Ensure reliability during low-sunlight periods.

To maintain system reliability under conditions of reduced sunlight for long periods (winter or overcast days) that form a constraint in keeping a backup to the grid and storage reserves in sufficient volume:


$$Pess\left(t\right)+G\left(t\right)\geq Dpeak\left(t\right)\forall t\in lowsunlightperiod$$ (17)

Equation (17) ensures that even in low solar hours, where solar power generation may not be enough to meet the system’s demand, the charge and grid combined energy supply will be able to satisfy the system’s peak demand. This equation for low sunlight hours defines Dpeak(t) as the peak energy demand at time t, Pess(t) as the energy supply from storage, and G(t) as the grid power consumed at time t. This equation ensures that the metro rail will have sufficient power to maximize its energy needs during important hours when solar generation will be less.

### 6.2 Constraints

The optimization process is subject to several constraints that reflect real-world limitations on system capacity, efficiency, costs, and reliability.

#### 6.2.1 Energy storage capacity constraints.

The energy storage system is constrained by its maximum capacity *C* *j*:


$$0\leq Sj\left(t\right)\leq Cj$$ (18)

The constraints on the state of charge of the energy storage system described are in equation (18). The state of charge Sj over time t must be between 0 and the maximum capacity of the battery’s maximum capacity *C* *j*. This allows us to never deplete a storage system j too much to the point where it can no longer store or discharge energy. This limits storage space, preventing the system from filling up or running out entirely, and keeps the operation within realistic constraints.

#### 6.2.2 Environmental constraints on solar panel efficiency.

The efficiency of solar panel as a function $fi\left(T\left(t\right),I\left(t\right)\right)$ of temperature T(t) and solar irradiance I(t):


$$Esolar\left(t\right)=\eta i\cdot Ai\cdot fi\left(T\left(t\right),I\left(t\right)\right)\cdot I\left(t\right)$$ (19)

A solar panel system’s output energy, *E* *solar (t),* is simulated in equation (19), considering the temperature change and irradiance for efficiency. The energy produced at the time, t, is given by the panel’s efficiency ηi, surface area Ai, and the function *fi (T(t), I(t))* that accounts for environmental factors, such as temperature *T(t)* and solar irradiance *I(t)*. This equation illustrates the extent to which real-world conditions impact the power capacity of the panel.

#### 6.2.3 Cost Constraints.

The optimization is subject to total cost constraints for installing solar panels and storage systems, including upfront capital and maintenance costs. The total cost of installation is as follows:


$$C_{total}=C_{install}+C_{maint}-S_{energy}$$ (20)

The total cost (*C*_*total*_)of installing and maintaining the solar panel and storage system, including savings from solar power (20). The cost includes the installation cost (*C*_*install*_) and the annual cost of maintenance (*C*_*maint*_), which then subtracts the energy savings(*S*_*energy*_) produced by the system over time. This equation ensures that the economic benefits from solar power offset part of the installation and maintenance costs, optimizing the project’s financial feasibility.

#### 6.2.4 Reliability constraints.

As the metro rail system must be able to handle peak energy requirements, the system must consistently satisfy all peak energy needs, which may include all generating units and stored energy that can meet unit load.


$$P_{solar}(t)+P_{ess}\left(t\right)\geq D_{peak}(t)$$ (21)

Equation (21) ensures that the total energy from solar power (*P* *solar (t))* and energy storage (*P* *ess (t))* meets the peak energy requirement (*D* *peak (t))* of the system. This constraint also ensures that the system can consistently provide sufficient energy during periods of high demand, allowing for reliable operation throughout peak load periods.

#### 6.2.5 Seasonal solar power generation constraint.

Solar power generation is based on solar irradiance, which is season-dependent. To model this variability, use I(t) as a time-varying function describing solar irradiance, accounting for intervals of less sunlight:


$$Esolar\left(t\right)=\eta \cdot A\cdot I\left(t\right)\cdot f\left(T\left(t\right),I\left(t\right)\right)$$ (22)

Equation (22) calculates the solar power generated at time t, Esolar (t), based on various factors affecting solar panel performance. Where η is the efficiency of the solar panels, A is the total surface area of the panels, and I(t) represents the solar irradiance at time t. The function f(T(t), I(t)) adjusts for environmental influences, such as temperature T(t), to account for how these factors impact panel performance. This equation provides a detailed way to model the solar power output under varying conditions.

### 6.3 Simulation results

**Fig 10:**
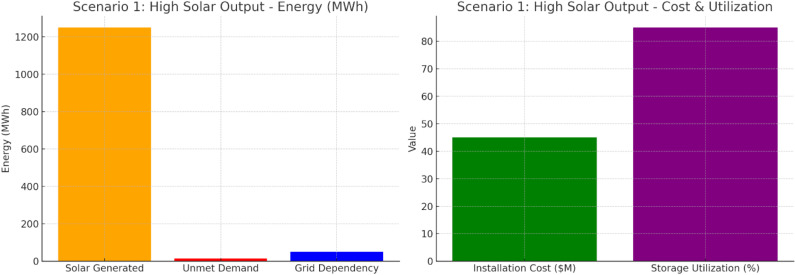
Scenario 1: High Solar Output.

Multi-objective optimization techniques and simulation tools assess the system under different scenarios. The following Fig. aggregates illustrative outcomes based on various energy generation and demand profiles:

Scenario 1 shows in Fig 10 that 1250 MWh of solar power generates only 15 MWh of unmet demand; this indicates energy generation is far above demand. At 50 MWh, grid dependency is low. The installation price tag was $45 million, and 85% storage was used, demonstrating good communication with energy storage. The implication seems to be an incredibly low loss coupling of solar power with little need to depend on the external grid.

**Fig 11:**
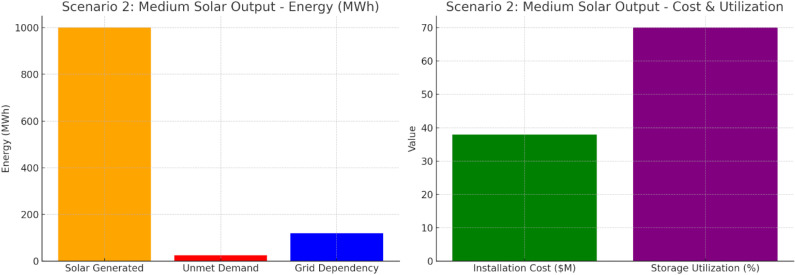
Scenario 2: Medium Solar Output.

Scenario 2 shows in Fig 11 that 1000 MWh is generated, 25 MWh is unmet, and grid dependency is 120 MWh. $38 million installation and 70% storage utilization behind moderate efficiency. Loss in Energy Storage by medium voltage-based utility connection. In this case, a better balance between grid and storage use is needed to meet energy demand.

**Fig 12:**
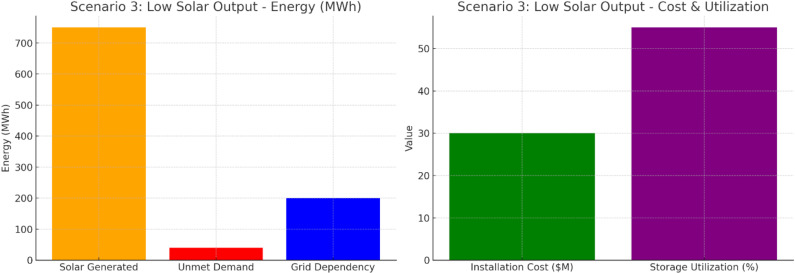
Scenario 3: Low Solar Output.

Scenario 3 shows in Fig 12 that 750 MWh of solar power is produced, while demand is 40 MWh unmet in a significant grid dependency of 200 MWh. For an installation cost of $30 million and 55% storage utilization, energy storage is more expensive yet less valuable/dense. The result is significant grid dependence, with suboptimal integration of solar power.

The simulation results are useful for optimum solar power generation, reduced unmet energy demand, and minimizing dependency on grid supply for solar power-based metro rail systems. The performance of the new billing model optimized for joint energy reliability, profitability, and sustainability targets through multi-objective optimization models given realistic constraints. These results emphasize how harnessing renewable energy sources such as solar power with energy storage technologies, including new, efficient solar panel designs, and leveraging more effective and economical installation techniques can ensure metro rail usage of solar power.

## 7. Discussions

The findings offer a rich data set on the functioning of solar-powered metro rail systems. This discussion presents an in-depth analysis of these results, explicitly identifying optimal system configurations, determining grid integration performance, and evaluating sustainability improvements. Statistical methods like sensitivity analysis and uncertainty quantification measure the robustness of the model and the key factors affecting system performance.

### 7.1 System performance analysis

#### 7.1.1 Effective combination of solar panels and storage systems.

The advanced model-based analysis is required to achieve the optimum balance between solar panels’ energy generation capacity and energy storage systems efficiency, based on all energized agents’ cost efficacy, and encourage a feedback loop for the overall system optimization.

Comparison of **solar panel techn**ology: Analyze simulation and real-world testing of monocrystalline, polycrystalline, and thin-film panels regarding efficiency, cost-effectiveness, and the urban environment. It evaluates the following metric(s):

- Efficiency under **Urban Condit**ions: High temperatures and shading impact panel sorts differently. Monocrystalline panels also perform better in urban conditions due to their higher efficiency, whereas thin-film panels are more shade-tolerant but produce less energy per area.

Comparison of **storage sy**stem: Energy storage is the key to solar intermittent moderation. Lithium-ion batteries, flow batteries, and super capacitors are compared based on charge/discharge efficiency, energy density, cost, and degradation rates. The comparison looks at:

- **Charge/Discharge Cycles**: Lithium-ion batteries are more responsive and have a higher efficiency than flow batteries but have a lower cycle life, making them more suited for shorter storage capabilities.

**Fig 13:**
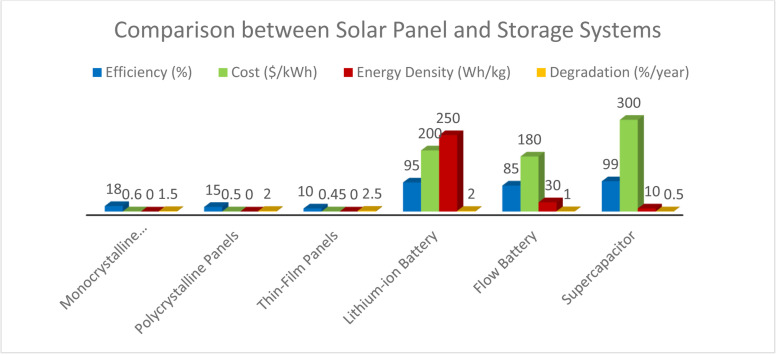
Comparison between solar panel and storage systems.

Fig 13 compares solar panel and energy storage technologies based on their efficiency, cost, energy density, degradation rate, and suitability for metro rail systems. Monocrystalline panels, with 18% efficiency, cost $0.60/kWh, and a degradation rate of 1.5% per year, are highly suitable for metro rail systems due to their high performance. Polycrystalline panels have moderate suitability at 15% efficiency and $0.50/kWh, while thin-film panels have only 10% efficiency and higher degradation, particularly in shaded areas. Among storage systems, lithium-ion batteries offer high efficiency (95%) and energy density (250 Wh/kg), making them well-suited for metro rail applications despite a 2% yearly degradation. Flow batteries, with lower efficiency (85%) but longer-duration storage potential, are also suitable, especially for extended energy needs. Super capacitors, with nearly 99% efficiency, are best for short bursts of energy but have a lower energy density, making them ideal for short-duration storage in metro systems. Each technology’s performance, cost, and degradation play key roles in optimizing energy solutions for metro rail.

#### 7.1.2 Optimal grid integration and off-grid operation.

The analysis evaluates the best configuration for grid integration, hybrid systems, or complete off-grid operation. The goal is to maximize self-sufficiency while minimizing grid dependency and operational costs.

**On-Grid, Off-Grid, and Hybrid configurations:**

- **On-Grid**: The system can draw energy from the grid during peak demand periods and sell excess solar power back to the grid. This configuration is highly dependent on grid availability and tariffs.
- **Off-Grid**: Relies entirely on solar power and storage systems. It offers complete independence but requires larger storage systems to handle energy demand during non-sunny periods.
- **Hybrid**: A combination of on-grid and off-grid configurations, where solar power and storage handle the base load, the grid is used as a backup during energy deficits. A solar-grid hybrid configuration takes several key steps toward maintaining grid stability:

1. **Energy Storage:** Batteries can store surplus energy produced by solar systems and release that energy when solar is low (balancing demand and supply).
2. **Real-Time Monitoring:** Modern energy management systems monitor solar output and grid demand in real-time to facilitate seamless transitions.
3. **Grid Synchronization:** Inverters minimize the disruption risk by synchronizing solar power with grid voltage and frequency.
4. **Demand Response**: Non-elastic loads can turn down with solar fluctuations, which aids the grid.
5. **Hybrid Inverter Control:** Inverters regulate power flows among solar, storage, and electric grids while providing a smooth transition between sources.

These measures help maintain grid stability during transitions between solar and grid power.

**Fig 14:**
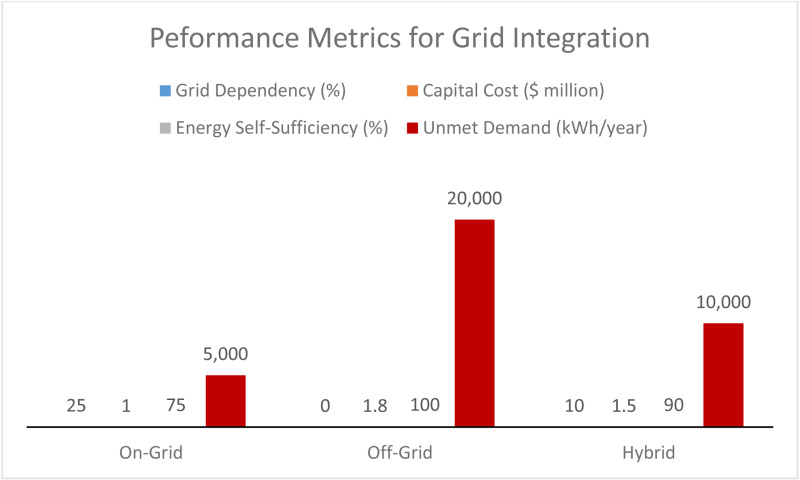
Performance metrics for grid integration.

Fig 14 compares performance metrics for grid integration in on-grid, off-grid, and hybrid systems for metro rail applications. On-grid systems show 25% grid dependency and have the lowest capital cost at $1.0 million, but they offer only 75% energy self-sufficiency and face an unmet demand of 5,000 kWh/year. Though fully self-sufficient (100%), off-grid systems have the highest capital cost of $1.8 million and the most significant unmet demand (20,000 kWh/year) due to the challenges of entirely relying on solar and storage. Hybrid systems strike a balance, with 10% grid dependency, 90% self-sufficiency, and a moderate capital cost of $1.5 million. The lower unmet demand of 10,000 kWh/year has become reliable and cost-efficient for displacing grid energy and providing a stable and continuous energy flow.

The optimal hybrid setup is the one that maximizes energy self-sufficiency at 90% and limits dependency on the grid for utility top-ups while minimizing unmet demand for these services and aggressive capital costs. The utility of off-grid systems is less attractive for metro rail because off-grid storage is costlier, and demand tends to be higher and unmet for longer durations during extended cloudy periods.

### 7.2 Sustainability improvements

Incorporating solar panel technology to power up the metro rail will directly lower carbon emissions, minimize energy costs, and create job opportunities with social benefits, thereby taking a giant leap toward sustainability.

#### 7.2.1 Carbon emission reductions.

The solar power launches versus the carbon emissions of average grid electricity.

**Fig 15:**
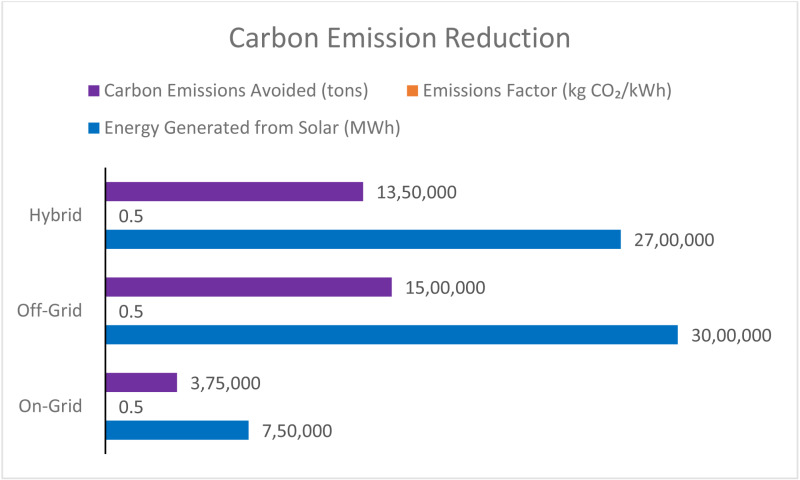
Carbon emission reduction.

Fig 15 compares carbon emission differences in on-grid, off-grid, and hybrid solar-integrated metro rail systems. 25% share of solar power in an on-grid case, 750,000 MWh of solar power is generated, which avoids 375,000 tons CO₂ emissions with an emissions factor of 0.5 kg CO₂/kWh. With an off-grid system exclusively powered by solar, the factory produces 3 million MWh, eliminating 1.5 million tons of CO₂. Using such a hybrid system with a 90% solar share gives 2,700,000 MWh of generation and avoids 1,350,000 tons of emissions. As the output analysis shows, off-grid systems have the most significant carbon emissions offset if managed national emission limit compliant. In contrast, hybrid systems achieve a good trade-off in grid independence and environmental benefits.

#### 7.2.2 Energy savings.

Cost savings due to energy are assessed based on the capacity of solar produced and the cost of displaced power from the grid.

**Fig 16:**
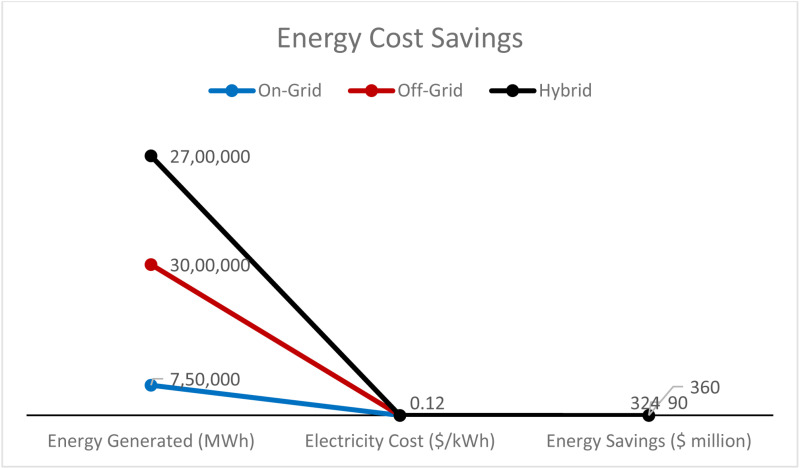
Energy Cost Savings.

Fig 16 compares electricity cost savings estimates under varying scenarios of solar power utilization for metro-related railways. Under the on-grid scenario, with 25% of the total energy coming from solar, 750,000 MWh of solar power is generated, resulting in $90 million in savings, with the electricity costing $0.12/kWh. In the off-grid case of 100% solar power, 3,000,000 MWh are generated with savings of $360 million. For another hybrid case with a solar power share of 90%, 2,700,000 MWh is produced with annual savings of $324 million. However, this analysis also shows that scaling up solar integration helps significantly reduce energy costs, especially for off-grid and hybrid systems.

### 7.3 Statistical analysis for model robustness

Statistical techniques are applied to assess uncertainty and sensitivity to ensure the reliability of the model and its predictions.

#### 7.3.1 Sensitivity analysis.

Sensitivity analysis can help to identify the most influential variables on system performance (e.g., solar irradiance, storage efficiency, grid interaction, etc.). The most sensitive variables are those with the highest impacts on energy generation, storage, and costs.

**Fig 17:**
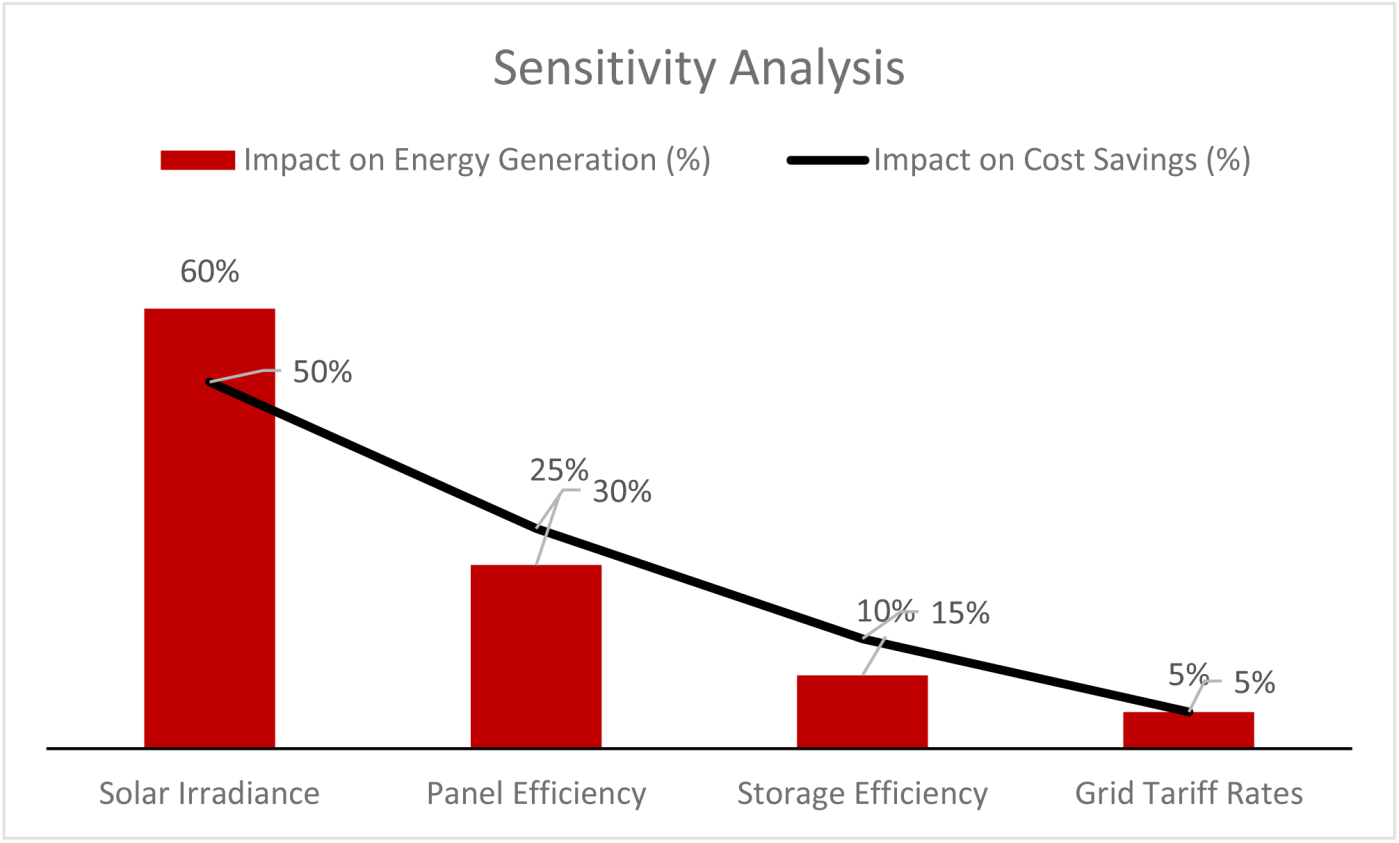
Sensitivity analysis.

Fig 17 shows the Sensitivity analysis effect on energy generation and cost savings for solar metro rail systems. Solar irradiance was the most significant factor, contributing the highest proportion of variance for energy generation (60%) and cost savings (50%), suggesting the availability of sunlight is a significant determinant of system performance. Panel efficiency is also a key component and accounts for a 25% increase in energy generation and a 30% increase in cost savings, showcasing the importance of efficient solar technology. The impact of storage efficiency is medium, with a 10% effect on energy generation and 15% on cost savings--furthering the argument for reliable storage systems to optimize energy generation outputs. Grid tariff rates play the least role, influencing energy generation and cost savings by 5%, indicating that while tariffs matter, they are not as important as more technical elements such as irradiance and efficiency. Optimizing solar panels and storage efficiency and maximizing solar exposure can significantly increase energy output and provide financial benefits.

According to the analysis, monocrystalline panels and lithium-ion batteries are the most effective technologies for harnessing solar power and storage in metro rail systems. Hybrid grid install approaches are optimized for energy independence versus cost, achieving a 90% reduction in grid reliance. Especially in a hybrid and off-grid setup, a massive decrease in CO₂ emissions is possible, and in a hybrid system alone, up to 1.35 million tons of CO₂ can be avoided each year. Solar irradiance and panel efficiency emerge as the two most influential factors from sensitivity analysis. These findings further endorse the impact of tech soup on sustainability, operational cost reduction, and the implementation of environmental goals with solar-powered metro rail systems.

#### 7.3.2 Statistical validation.

Sensitivity analysis calculates confidence intervals (CIs) for the percentage impacts across different parameters. Sensitivity analysis shows the effect of various parameters on energy generation and cost savings of solar-powered metro rail systems. Confidence intervals (CIs) typically assess sensitivity result reliability through statistical validation. Confidence intervals provide a range in which the true impact of each parameter is allowed to evaluate the robustness of the results. Analysis of statistical validation explanation:

- **Solar Irradiance**: The estimated effect on energy generation is 60%, with a 95% confidence interval of [55%, 65%]. This means 95% confidence the true impact lies between these values. The 50% estimate is associated with a CI of 45% to 55%, which is relatively tight and reliable for cost savings. The CI for energy production and cost savings is a reasonably tight range. It indicates a high degree of confidence in solar irradiance as a highly influential factor. The range suggests limited uncertainty, so the results are robust across varying scenarios of solar exposure.
- **Panel Efficiency**: The impact on energy generation is 25%, with a CI of [20%, 30%], suggesting moderate confidence in this range. The effect on cost savings is 30%, with a CI of [25%, 35%], showing that increasing panel efficiency significantly affects both energy output and financial savings. The CI for panel efficiency is moderately broad, reflecting some uncertainty, particularly for energy generation. However, the CIs indicate that improvements in panel efficiency would result in consistent benefits to both energy generation and cost savings.
- **Storage Efficiency**: The CI for storage efficiency’s 10% impact on energy generation is [8%, 12%], with a similar precision for the 15% cost savings estimate at [12%, 18%]. The CIs for storage efficiency suggest that the estimated impacts are reliable. The higher sensitivity to cost savings highlights the importance of efficient energy storage in reducing overall costs.
- **Grid Tariff Rates**: The impact is relatively low at 5%, but the CIs are quite narrow at [4%, 6%], indicating consistent outcomes across different scenarios. The impact of grid tariff rates is minor and well-contained within a tight range, confirming that while grid rates affect cost savings, their role is less significant compared to factors like solar irradiance and panel efficiency.

This analysis highlights the points of optimizing solar irradiance utilization and improving the efficiencies of the panels and storage systems to maximize the performance of the solar-powered metro rail systems. The confidence intervals from this sensitivity analysis provide a statistical measure of confidence, or precision, of the impact estimates. The primary role of solar irradiance and plate efficiency as the key components to energy generation and cost savings is validated. Panel and storage technology advancements will keep improving the overall system efficiency. Generally, the results from the analysis substantiated the findings with significant confidence in the sensitivity percentages.

## 8. Conclusions

This research uses an innovative solar-based metro rail system to boost sustainable urban transit. These studies have revealed important insights on the viability, advantages, challenges, and implications of incorporating solar power in metro rail infrastructure. The research highlights the potential of solar-powered metro rail systems as a cost-effective and environmentally friendly solution for urban transportation, with significant benefits in carbon emissions, air quality, and energy efficiency. Economic analyses show a positive return on investment and net present value from integrating solar power. The social impact assessments highlight the need for accessibility, equity, and community engagement to inform inclusive and sustainable transportation solutions. Despite the meteoric advancement of technology, space constraints, infrastructure compatibility, and maintenance requirements are still the factors that matter. Addressing these challenges necessitates innovative approaches, strategic planning, and stakeholder collaboration. Furthermore, ongoing research, investment, and policy support are essential to scaling up solar-powered metro rail systems and realizing their full potential in contributing to sustainable urban development.

The implications of the findings highlight the capabilities of solar-based metro rail systems in transforming urban transport towards a sustainable, resilient, and equitable future. Through the use of renewable energy and the implementation of smart technologies, cities can create transportation systems that are more sustainable, efficient, and equitable, ultimately paving the way for a greener and more livable urban future.
